# Interventions to mitigate vaping misinformation: protocol for a scoping review

**DOI:** 10.1186/s13643-022-02094-0

**Published:** 2022-10-09

**Authors:** Navin Kumar, Sam Hampsher, Nathan Walter, Kate Nyhan, Munmun De Choudhury

**Affiliations:** 1 Yale School of Medicine, New Haven, CT USA; 2 BOTEC Analysis, LLC, Woodland Hills, CA USA; 3 Northwestern University, Evanston, IL USA; 4 Harvey Cushing/John Hay Whitney Medical Library, Yale University, 333 Cedar Street, New Haven, CT 06520-8014 USA; 5 Department of Environmental Health Sciences, Yale School of Public Health, New Haven, CT USA; 6 School of Interactive Computing, Georgia Tech, Atlanta, GA USA

**Keywords:** Vaping, Misinformation, Health, E-cigarette, Mitigate

## Abstract

**Background:**

The impact of misinformation about vapes’ relative harms compared with smoking may lead to increased tobacco-related burden of disease. To date, no systematic efforts have been made to chart interventions that mitigate vaping-related misinformation. We plan to conduct a scoping review that seeks to fill gaps in the current knowledge of interventions that mitigate vaping-related misinformation.

**Methods:**

A scoping review focusing on interventions that mitigate vaping-related misinformation will be conducted. We will search (no date restrictions) MEDLINE, Scopus, EMBASE, CINAHL, PsycINFO, Web of Science Core Collection, Global Health, ERIC, and Sociological Abstracts. Gray literature will be identified using Disaster Lit, Google Scholar, Open Science Framework, governmental websites, and preprint servers (e.g., EuropePMC, PsyArXiv, MedRxiv, JMIR Preprints). Study selection will conform to Joanna Briggs Institute Reviewers’ Manual 2020 Methodology for JBI Scoping Reviews. Only English language, original studies will be considered for inclusion. Two reviewers will independently screen all citations, full-text articles, and abstract data. A narrative summary of findings will be conducted. Data analysis will involve quantitative (e.g., frequencies) and qualitative (e.g., content and thematic analysis) methods. Where possible, a single effect size of exposure to the mitigation of vaping-related misinformation will be calculated per sample.

Similarly, where possible, each study will be coded for moderating characteristics to find and account for systematic differences in the size of the effect or outcome that is being analyzed. Quality will be appraised with the study quality assessment tools utilized by the National Heart, Lung, and Blood Institute.

Findings will be subjected to several different publication bias tests: Egger’s regression test, Begg and Mazumdar’s ran correlation test, and generation of a funnel plot with effect sizes plotted against a corresponding standard error.

**Discussion:**

Original research is urgently needed to design interventions to mitigate vaping-related misinformation. The planned scoping review will help to address this gap.

**Systematic review registration:**

Open Science Framework osf/io/hy3tk.

**Supplementary Information:**

The online version contains supplementary material available at 10.1186/s13643-022-02094-0.

## Background

The recent introduction of alternative forms of nicotine products into the marketplace (e.g., e-cigarettes, heated tobacco products, and smokeless tobacco) has led to a more complex informational environment. For example, misinformation from the online marketing of vapes (e-cigarettes) by manufacturers, retailers, and social media influencers has claimed that e-cigarettes contain only water vapor and are harmless [1]. Misinformation is defined as information that has the features of being false or clearly unsubstantiated, determined based on expert opinion and evidence [2].

Misinformation may downplay the risks of vape use and may be in part responsible for the recent youth vaping epidemic [3–6]. Conversely, online misinformation that vapes are just as or more harmful than smoking [5, 6] may deter current cigarette smokers who are unable to quit smoking from considering reducing harms by switching to vapes as a tool to be used for a period of time to assist in achieving abstinence from cigarettes [7]. For example, regarding the outbreak of vaping-related lung injury (EVALI), most cases were related to the consumption of vitamin E acetate, an additive included in some tetrahydrocannabinol devices [8]. However, news reports did not always differentiate between tetrahydrocannabinol devices and standard nicotine-based vapes [9, 10], perhaps disproportionately characterizing vaping harms. Thus, the impact of misinformation about vapes’ relative harms compared with smoking may lead to increased tobacco-related burden of disease [11].

While there have been studies on preventing vaping among adolescents [12, 13], and the effect of vaping misinformation on attitudes toward vapes [1], and vaping misinformation more broadly [10, 14, 15], there is limited research on interventions to mitigate misinformation about vapes. Thus, we are far from knowing when and how to intervene best. Regarding mitigating misinformation, we refer to identifying places where misinformation could have an impact on behavior and blocking those conduits, and/or identifying causal pathways for behavior change and developing interventions that reduce the impact of misinformation. As a clarification, the scientific consensus is that vape aerosol contains fewer numbers and lower levels of toxicants than smoke from combustible tobacco cigarettes [16]. However, the use of vapes results in dependence on the devices, but with apparently less risk and severity than that of combustible tobacco cigarettes [16].

To date, no systematic efforts have been made to chart interventions that mitigate vaping-related misinformation. Past reviews have detailed interventions to mitigate misinformation on social media [17–20], the prevalence of misinformation on social media [21, 22], and whether messages about vapes alter harm perception and behavioral intentions [23], but have not focused on vaping-related misinformation. To provide information that can be used to design effective interventions for vaping misinformation, we plan a scoping review that seeks to compile published evidence in the field to identify gaps in the current understanding of experimental evidence regarding the mitigation of vaping-related misinformation.

We will conduct a scoping review rather than use other methods of research synthesis because scoping reviews are appropriate for mapping an area of research [24]. The review will take a broad view of vaping misinformation, from claims that vaping is completely safe to statements around vaping being more dangerous than combustible cigarettes. We will consider both explicit misinformation (information that is verifiably false based on current scientific evidence) and implicit misinformation (information that misleads the public about the harms and benefits of vaping, e.g., inaccurate and incomplete information) [25], where the primary audience for misinformation is the general public.

## Methods/design

The review protocol has been preregistered within the Open Science Framework database (osf/io/hy3tk). It is being reported in accordance with the reporting guidance provided in the Preferred Reporting Items for Systematic Reviews and Meta-analyses extension for Scoping Reviews (PRISMA-ScR) [26] (see checklists in Additional file 1). Research objectives, inclusion criteria, and methodological techniques will be determined before study commencement using the Joanna Briggs Institute Reviewers’ Manual 2020 Methodology for JBI Scoping Reviews [27]. This process will adhere to the indicated framework: (1) identifying research question, (2) developing a comprehensive search strategy, (3) identifying relevant studies, (4) selecting studies, (5) charting data, and (6) collating, summarizing, and reporting results. The study team will develop a search strategy as recommended by the 2020 Methodology for JBI Scoping Reviews.

This scoping review will be conducted by five individuals: four researchers from several universities worldwide, from a range of disciplines (e.g., public health, communication studies, nursing, medicine, political science, computer science), and an informationist from the Harvey Cushing/John Hay Whitney Medical Library at Yale University. The objective of the scoping review is to develop a better understanding of the current research landscape around interventions to mitigate vaping-related misinformation by investigating existing studies and gaps in the research. The broad research questions are “what has been reported on interventions to mitigate vaping misinformation?” and “what are the gaps in the current knowledge base on interventions to mitigate vaping misinformation?” The search strategy will be performed in line with techniques that enhance methodological transparency and improve the reproducibility of the results and evidence synthesis.

### Information sources and search strategy

The primary source of literature will be a structured search of electronic databases (no date restrictions): MEDLINE, Scopus, EMBASE, CINAHL, PsycINFO, Web of Science Core Collection, Global Health, ERIC, and Sociological Abstracts. The secondary source of potentially relevant material will be a search of preprint servers (e.g., EuropePMC, PsyArXiv, JMIR Preprints), Google Scholar (e.g., the first five pages will be searched based on guidance in prior research), Open Science Framework, and governmental websites. The references of included documents will be hand-searched to identify any additional evidence sources. We will also conduct forward citation chaining. The search strategy will be designed by a research librarian and peer reviewed by using the Peer Review of Electronic Search Strategies (PRESS) checklist [28]. A draft search strategy for Scopus is provided in Additional file 2. We will use search terms similar to our main search to find articles for inclusion. The same keywords for the main search will be used to search gray literature each time. All gray literature will be compiled in a folder and reviewed similarly to articles obtained from our database searches. EndNote, a bibliographic software, will be used to store, organize, and manage all references [29].

### Eligibility criteria

We will include all intervention studies to mitigate vaping misinformation including inaccurate and incomplete information, where the primary audience for misinformation is the general public. Only English language studies will be considered for inclusion. Past work indicated that excluding non-English language records from a review seemed to have a minimal effect on results [30, 31].

#### Inclusion criteria

Published research (peer reviewed and gray literature where primary data was collected such as reports, research letters, and briefs) investigating interventions to mitigate vaping misinformation (as long as the authors have denoted the topic of study as misinformation) in all populations and settings will be eligible for inclusion.

Only intervention-based studies will be included (e.g., experimental studies, quasi-experimental studies, randomized controlled trials).

There will be no restrictions on region.

Studies reported only as conference abstracts will also be included, only if we do not have access to the full paper. Conference abstracts are often left out of systematic reviews as they may not contain adequate information to conduct quality assessment or a meta-analysis. Here, we will include conference abstracts as they are often published earlier than full manuscripts [32], which is key to a thorough scoping review on an ongoing phenomenon.

#### Exclusion criteria

Commentaries, correspondences, case reports, case series, editorials, and opinion pieces will be excluded. Case reports and case series often contain relatively limited evidence [33].

Qualitative studies will be excluded.

Non-intervention studies will be excluded.

Governmental, other agency guidelines and white papers will be excluded. Reviews such as systematic reviews and scoping reviews will be excluded but we will review the references in these for inclusion, if applicable.

### Screening and selection procedure

All reports identified from the searches will be screened by two reviewers independently. First, titles and abstracts of articles returned from initial searches will be screened based on the eligibility criteria outlined above. Second, full texts will be examined in detail and screened for eligibility. Third, references of all considered articles will be hand-searched to identify any relevant report missed in the search strategy. We will also conduct forward citation chaining. Any disagreements will be resolved by discussion, or if necessary, with a third reviewer. A flow chart showing details of studies included and excluded at each stage of the study selection process will be provided. We will contact authors where necessary if the abstracts do not provide sufficient information [32]. Covidence will be used to manage the title/abstract and full-text screening phases [34].

### Data extraction

Reviewers will undergo a practice exercises till they have a high level of agreement (> 0.8 kappa) and then independently extract data from studies. Reviewers will abstract the data using a pretested data extraction template. We will use a standardized coding protocol to collect information such as title of study, authors, date published; study setting; study design; description of methodology; description of the study sample; type of intervention; type of misinformation (if any); and main findings. We will note which studies are preprints and thus have not been formally peer reviewed. We will also note if some studies fail to report appropriate information.

### Data synthesis

Outcomes and other information collected regarding selected studies will be synthesized using quantitative (e.g., outcomes) and qualitative (e.g., content and thematic analysis) methods, with a narrative summary of findings conducted. Synthesis will be presented in tables, summary data in graphs, and individual data for each study in tables. The broad goal of the synthesis is to identify gaps in research and present recommendations for future research agendas. A single effect size of exposure to the mitigation of vaping-related misinformation will be calculated per sample, where possible. Where studies reported several relevant outcomes, all effect sizes will be recorded and then averaged into a single outcome [35]. Similarly, where studies employed multiple types of mitigation in the same study, all relevant effect sizes will be retrieved from the study and then averaged for the analysis [36]. Each study will be coded for moderating characteristics. These characteristics will be used to conduct moderation analysis. Moderation analysis is a technique to find and account for systematic differences in the size of the effect or outcome that is being analyzed. To safeguard against potential violations of independence of effect sizes, all moderators will be coded at the level of the study [37].

### Quality and bias assessment

Study quality will be appraised with the study quality assessment tools utilized by the National Heart, Lung, and Blood Institute. Findings will be subjected to several different publication bias tests: Egger’s regression test [38], Begg and Mazumdar’s ran correlation test [39], and generation of a funnel plot with effect sizes plotted against a corresponding standard error. Heterogeneity will be assessed with Cochrane’s *Q* and *I*^2^ statistics. To explore potential causes of significant heterogeneity, we will undertake a *Q*-test subgroup analysis.

## Discussion

There has been limited research which compiles available evidence from various settings around interventions which mitigate vaping misinformation. Our review will provide an overview of these studies, synthesizing evidence. We will provide an overview of known gaps in the literature, such as how to target corrective information better and to make it more effective, disrupt the formation of linkages between group identities and false claims, and reduce the flow of cues reinforcing those claims from elites and the media [40].

There is much anecdotal work around misinformation and vaping, with few intervention studies. The planned review will highlight areas of research focus and gaps which require more attention. Results will provide high-level information to inform, support, and customize design of interventions to mitigate vaping misinformation. As researchers attempt to minimize vaping misinformation, they need to be aware of scientific evidence to develop interventions to achieve their aim. The planned scoping review seeks to provide this evidence by contributing an evaluation of what is currently known about interventions to mitigate vaping misinformation, with the goal of identifying gaps in research and presenting recommendations for future research foci.

The methodological strength of the planned scoping review is the use of a transparent and reproducible procedure for a scoping literature examination. We state the data sources, search strategy, and data extraction [41]. Through publishing this research protocol, we strengthen the clarity of the search strategy. This protocol can be applied to other substances or public health topics, providing insight for conducting similar reviews. Any amendments to this protocol will be documented in the final published scoping review with reference to saved searches and analysis. Results of the review will be disseminated in a peer-reviewed journal and likely in other media such as conferences, seminars, and symposia. The protocol and final review article will be made open access upon publication. As per PRISMA-ScR guidelines, we will present results in a user-friendly format [42].

### Limitations

Our planned review should be read in line with some limitations. Although we plan to search several databases and gray literature sources, we may miss some studies. Our explicit focus on misinformation may miss articles around health communication, risk communication, advertising, marketing, and packaging. Not all authors we reach out to may respond and we may thus miss some unpublished work.

There is a strong chance that many of the primary studies identified will be of highly variable quality and many may operate on a flawed assumption that the presence of misinformation online is equivalent to the impact of misinformation on behaviors. A scoping review may not be able to tell the difference between articles that include correctly measured behavior outcomes and articles that act only on the source of misinformation without considering exposure and influence. Misinformation is an inherent moving target and as advancements are made in our knowledge and new research results surface as does the context of what is considered accurate; nonetheless, it is important to understand how misinformation is mitigated to meet advancements in science.

## Supplementary Information

**Additional file 1.** Preferred Reporting Items for Systematic reviews and Meta-Analyses extension for Scoping Reviews (PRISMA-ScR) Checklist.

**Additional file 2.** MEDLINE draft search strategy.

## Data Availability

The datasets used and analyzed will be made available upon reasonable request.

## Abbreviations

PRISMA-ScR: PRISMA Extension for Scoping Reviews
JBI: Joanna Briggs Institute
COVID-19: Coronavirus disease 2019
