# Supplementary material for: Interventions to mitigate vaping misinformation: protocol for a scoping review
Source: Syst Rev. 2022 Oct 9;11:214. doi: 10.1186/s13643-022-02094-0 (PMC9548303; doi:10.1186/s13643-022-02094-0)
Supplement: Supplementary file 2 — Additional file 2. MEDLINE draft search strategy. [file 13643_2022_2094_MOESM2_ESM.docx]

# MEDLINE DRAFT SEARCH STRATEGY

**Search strategy**

(TITLE-ABS-KEY(misinformation OR disinformation OR ”conspiracy theor*” OR literacy OR literate OR correct* OR retract* OR rumor* OR rumour* OR truthful* OR ”fake news” OR fact-check* OR ((information OR advertis* OR marketing OR packaging ) W/3 ( verified OR unverified OR credib* OR accura* OR inaccura* OR trust* OR misleading OR quality OR false ) ) OR infodemic OR pseudoscien* OR pseudo-scien* OR conspiratorial OR gossip OR hoax* OR ”urban legend*” OR myth* OR fallac* OR falsehood* )) AND

(TITLE-ABS-KEY( vape* OR vaping OR e-cig* OR ”electronic cigarette*” OR ”electronic nicotine” OR ”alternative nicotine” OR ”electronic vapo*” OR

e-vapo* OR ( ( tobacco OR nicotine ) AND nebuli* ) OR ”heated tobacco product*” OR e-smoking ) )
